# Effectiveness of avocado leaf extract ( *Persea americana* Mill.) as antihypertensive

**DOI:** 10.12688/f1000research.124643.2

**Published:** 2023-11-13

**Authors:** Dwi Sutiningsih, Dewi Puspito Sari, Mateus Sakundarno Adi, Mochammad Hadi, Nur Azizah Azzahra

**Affiliations:** 1 Master of Epidemiology, School of Postgraduate, Diponegoro University, Semarang City, 50241, Indonesia; 2 Department of Epidemiology and Tropical Diseases, Faculty of Public Health, Diponegoro University, Semarang City, 50275, Indonesia; 3 Department of Ecology and Biosystematics, Department of Biology, Faculty of Science and Mathematics, Diponegoro University, Semarang City, 50275, Indonesia

**Keywords:** Avocado leaf extract, Persea americana Mill, Antihypertension, Hypertension, Systolic Blood Pressure (SBP), Diastolic Blood Pressure (DBP)

## Abstract

Background

Avocado leaves ( *Persea americana* Mill.) have been traditionally used as antihypertensive. Various studies have tested the benefits of the *Persea americana* Mill. in treating hypertension but still using extracts not yet using nanoparticles. Therefore, this study aims to study the effectiveness of avocado leaf extract, including the nanoparticles, on salt-induced hypertensive Wistar rats in inhibiting the ACE enzyme, which causes decreasing systolic blood pressure (SBP) and diastolic blood pressure (DBP) as well as increasing urine volume.

Methods

This study used an experimental *in vivo* study design involving 24 white male Wistar rats ( *Rattus norvegicus*), aged 2–3 months, weighing 130–250 g, and of a healthy condition with active movement. The samples were randomly divided into six treatment groups and post-test only research design with control group design.

Results

The result of the study showed that the extract contained a quercetin of 1129.597 ppm and 10 mineral compounds, including potassium, with the highest content. The analysis of antioxidant activity using the 2,2-diphenyl-1-picrylhydrazyl method showed that the antioxidant activity at IC _50_ was 44.734 ppm. The *P. americana* leaf extract nanoparticles had the most significant result in reducing blood pressure in Wistar rats induced by 16% NaCl for 14 days SBP to normal was by 68.75 mmHg (175.00±21.57 mmHg to 106.25±9.21 mmHg) and 55.25 mmHg (128.42±25.75 mmHg to 73.17±15.75 mmHg), respectively. Also, avocado leaf extract caused the decrease of SBP from 164.92±7.16 mmHg to 116.83±6.36 mmHg and DBP from 118.42±16.24 mmHg to 82.83±6.47 mmHg. The *P. americana* leaf extract nanoparticles resulted in the most urine volume at 13.5 ml, with the highest DAI value of 2.25. The ACE inhibitor test using the ELISA method showed that the *P. americana* leaf extract and the nanoparticle extract inhibition against the ACE enzyme were 60.0±12.1 % and 59.5±3%, respectively. The mean serum nitrate as a result of *P. americana* leaf extract (K4) and nanoparticles of *P. americana* leaf extract (K5) were 44.0±9.0 μmol/L and 41.1±11.5 μmol/L, respectively. The mean of serum nitrite was led by *P. americana* leaf extract (K4), and nanoparticles of P. americana leaf extract (K5) were 83.7±24.0 μmol/L and 81.0±23.2 μmol/L, sequentially.

Conclusions

The study showed that the leaf extract and nanoparticle extract of *Persea americana* Mill. was effective as an antihypertensive.

## Introduction

Hypertension is an increase in blood pressure measured twice in resting conditions, with systolic blood pressure (SBP) and diastolic blood pressure (DBP) of >140 mmHg and >90 mmHg in adults, respectively. ^1^ Various risk factors or their interactions lead to hypertension, such as obesity, unhealthy diet (excessive sodium salt, low potassium diet, and excessive intake of saturated fatty acids), heavy alcohol consumption, tobacco use, genetics, physical inactivity, persistent stress, and unhealthy environments. ^2^ ^,^ ^3^

Hypertension is a cardiovascular risk factor that causes many deaths worldwide. Chronic hypertensive conditions can lead to kidney failure, stroke, and ischemic heart disease. ^1^ ^,^ ^4^ ^,^ ^5^

Recent basic health research (Riskesdas) reported that the prevalence of hypertension in Indonesia increased from 25.8% in 2013 to 34.11% in 2018. ^6^ A chronic hypertensive state may lead to complications; thus, hypertension should be managed and treated properly. Management of hypertension is essential because high blood pressure is also a risk factor for several diseases. ^1^ Treatment includes pharmacological and non-pharmacological methods. ^7^ However, pharmacologically, prolonged use of drugs such as diuretics, calcium channel blockers, angiotensin receptor blockers, angiotensin-converting enzyme (ACE) inhibitors, and beta-blockers can cause side effects. The body may not be able to tolerate the appropriate drug levels. The drugs cannot completely cure the disease, so the drugs must be maintained for long periods. Moreover, hypertension drugs have high economic value because they must be purchased regularly over the course of the patient’s life. ^8^

In Indonesia, Malays utilize diverse plant species that have shown effectiveness in treating various diseases. For example, residents of the Jambi Province, Indonesia, treat hypertension using natural ingredients that have been processed into traditional medicines, such as avocado leaves ( *Persea americana* Mill.). Malays switched from using chemical drugs to herbal medicines because of the belief that traditional medicines that come from nature are easily tolerated by the body, have low economic value, and have relatively high safety despite long-term use. *P. americana* leaves are considered an effective antihypertensive agent because they are rich in flavonoids and quercetin compound, which are considered effective in reducing high blood pressure. ^5^ ^,^ ^7^ ^,^ ^9^ ^–^ ^11^

Various studies in hypertensive rats have shown that quercetin can exert a diuretic effect by increasing urine volume, thereby decreasing blood pressure. ^12^ ^,^ ^13^ Antihypertensive therapy using quercetin compound administered continuously can inhibit conversion of ACE from angiotensin I to angiotensin II, which causes vasoconstriction in blood vessels and consequently hypertension. ^5^ ^,^ ^7^ ^,^ ^14^ ^,^ ^15^ Inhibition of ACE, along with the increase in nitric oxide and nitrate oxide levels, inhibits oxidative stress due to decreased levels of antioxidants. ^15^ ^–^ ^17^

The Wistar rat, which has been bred at the Wistar Institute since 1906, is one of the most widely used animal model in biomedical research. ^13^ Rats are mammals; therefore, the treatment response may be similar to that of other mammals. The use of rats as experimental animals is also based on economic considerations, and that the rat’s life span is only 2-3 years with a reproduction time of 1 year. The advantages of white rats over wild rats are that they mature quickly, do not show seasonal mating, and reproduce faster. Other advantages of using a laboratory animal include that it is effortless to handle, it can be left alone in a cage as long as it can hear the sounds of other mice, and it is large enough to facilitate observation. ^18^

A previous study by Auwal *et al.* (2017) demonstrated that increasing the *in vivo* efficacy of antihypertensive biopeptides of chitosan nanoparticles using the ionic gelation method in spontaneously hypertensive rats proved that the angiotensin converting enzyme (ACE) inhibitory biopeptide stabilized by chitosan nanoparticles effectively reduced blood pressure for a long period. ^19^ Mariangela’s (2011) research related to a new formulation in the treatment of hypertension proved that the nanoparticle method was effective as an antihypertensive in the kidney, heart, or smooth muscle organs. ^20^ The results of Yuan’s (2012) study revealed that nanoparticles have better properties for pharmacokinetic drugs *in vivo* because the nanoscale size can help penetrate tissue through capillary blood vessels and epithelial layers. ^21^Another study by Sun’s (2014) showed that nanoparticles were durable and had a significant antihypertensive effect on spontaneously hypertensive rats. ^22^

Various studies used the extract of *P. americana* leaves in to investigate its effectiveness in treating hypertension. However, the previous studies did not assess the effect of nanoparticles extract. ^5^ ^,^ ^9^ ^,^ ^10^ ^,^ ^23^ ^,^ ^24^ The number of hypertension cases is increasing worldwide, ^25^ and there is a need for information regarding herbal medicine to treat hypertension. Therefore, this study is relevant nowadays and, in the future, to provide information regarding hypertension treatment. Moreover, there is a need to examine differences in the effectiveness of modern and herbal antihypertensive medicines *in vivo* through measuring the reduction in SBP and DBP and increase in urine volume. Thus, this study aimed to investigate the antihypertensive effects (such as inhibition of ACE, decreasing SBP, decreasing DBP, as well as increasing urine volume and increase in nitric oxide and nitrate oxide levels) of *P. americana* leaf extracts and nanoparticles *in vivo* involving male Wistar rats to develop potential natural materials of *P. americana* leaves in an attempt to control the prevalence of hypertension, especially in Sarolangun Regency, Jambi.

## Methods

### Materials

This study was conducted *in vivo* on white male Wistar rats ( *Rattus norvegicus*) aged 2–3 months, weighing 130–250 g, and of a healthy condition with active movement. Wistar rats that died during the acclimatization were excluded. There were 30 Wistar rats initially but during the period, six died. Thus, 24 Wistar rats were included in the sample. Wistar rats were placed in a cage at room temperature ranging from 25 to 28°C, with husks for animal rearing. The rats were fed standard food with BR-II pellets and were given sufficient distilled water. The health of Wistar rats was monitored every day with the general assessment of animal activity, food, and water intake, as well as by weighing rats on day 0 and day 15. Several rats experienced stress when placed in metabolic cages to measure urine volume because the rats were not adapted first. These rats were immediately fed and given distilled water.

Test animals were obtained from the Laboratory of Pharmacology and Toxicology, Section of Pharmacology and Clinical Pharmacy, Faculty of Pharmacy, University of Gadjah Mada (UGM). *P. americana* leaves were acquired from Bandungan Subdistrict, Semarang Regency, Central Java Province, Indonesia. Extracted nanoparticles were made from 2% liquid chitosan biopolymer at pH 4, which was obtained from the Faculty of Pharmacy, UGM. Sodium tripolyphosphate and 92% acetic acid were obtained from the chemical store “Utama Sari”. The modern medicine used as a comparison was furosemide (phytopharmaca), which was obtained from K24 pharmacy in Yogyakarta, and 16% NaCl solution and 0.5% carboxymethyl cellulose solution were obtained from Faculty of Pharmacy, UGM. The other chemical materials were 70% ethanol, distilled water, and 2.5 g sodium carboxymethyl cellulose.

### Study design

This experimental *in vivo* study used 24 white male Wistar rats which were divided into six groups with four Wistar rats in each group using a simple random sampling method. Calculation of the number of samples in each group based on the Federer formula: ^26^

$$\left(t-1\right)\left(n-1\right)>15$$

Formula description:

t: Number of experimental groups

n: Number of samples in each group

Based on the calculation, the number of samples in each group was four; therefore, all total samples were 24. Moreover, this study employed post-test design and a control group. The extract was made following the maceration method with 70% ethanol solvent. Each nanoparticle of *P. americana* leaf extract was made of a chitosan biopolymer and encapsulated by absorbing the active compounds in the extract. The following parameters were assessed: quercetin levels, antioxidant activity, mineral compound, changes in SBP, DBP, urine volume, DAI, increased urine volume, ACE inhibitions, nitrite oxide, nitrate oxide levels, SBP differences between groups, differences in DBP between groups, and urine volume test.

This study observed the variables consisting of independent variables, confounding, and dependent variables. The independent variables were the leaf extract of *P. americana* Mill and its nanoparticle preparations, the confounding variable was particle size, and the dependent variables were a decrease in SBP and DBP, and an increase in urine volume.

### Ethics

This study has obtained permission from the Health Research Ethics Committee (KEPK) of the Faculty of Public Health University of Diponegoro (UNDIP; No. 121/EA/KEPK-FKM/2019, dated May 15, 2019).

### Determination of avocado leaves ( *P. americana*)

Before the extract and nanoparticles of *P. americana* leaves were manufactured, avocado plants were examined at the Laboratory of Ecology and Biosystermatic, Faculty of Science and Math, Diponegoro University (UNDIP).

### Phytochemical screening

Extracts were obtained in solid preparations subjected to phytochemical tests to determine levels of quercetin, antioxidant activity, and mineral compounds.

### Creation of rat hypertension models

To minimize unexpected potential confounders, first, the animals were acclimatized for three days to the conditions of the experiment to avoid stress before the treatment, placed in the same room temperature cage ranging from 25 to 28°C, and given the same standard food of BR-II pellets and sufficient distilled water. Then, 24 white male Wistar rats were orally given 3 mL of 16% NaCl solution per day for 14 days to attain above-normal blood pressure. After 14 days, DBP and SBP were measured by the tail cuff method using *CODA*™ mouse rat tail-cuff system. The cuff on the tail was inflated until the SBP was above normal and the pulse disappeared, before the cuff pressure was slowly reduced. When the DBP is low, the pulse reappears. This measurement method is in accordance with blood pressure measurement using a sphygmomanometer in humans.

### Dosage determination

The dose of *P. americana* leaf extract, which significantly reduced blood pressure in Wistar rats, was 100 mg/kg body weight (BW). ^9^ In this study, six treatment groups were created. Four investigators (DS, DPS, MSA, MH) were aware of the group allocation. The first and second investigators (DS and DPS) were responsible for the allocation, experiment conduct, outcome assessment, and data analysis. The third and fourth investigators (MSA, MH) were responsible for the outcome assessment and the data analysis.

Group 1 (K1) was the normal control group administered with 3 mL/100 g BW distilled water per day. Group 2 (K2) was the negative control group administered with 3 mL of 16% NaCl per day. Group 3 (K3) was the positive control group in which rats were orally given a one-time 40 mg furosemide (phytopharmaca) suspension at a dose of 1.008 mg/200 g BW, similar to the usual dose of furosemide in humans. The dose in mice was 5.04 mg/kg BW, equivalent to 2 mL. Group 4 (K4) was the test group that received extract of *P. americana* leaves administered at a dose of 100 mg/kg BW with 2 mL suspended in carboxymethyl cellulose and 0.5% NaCl solutions. Group 5 (K5) was the test group treated with chitosan nanoparticles of *P. americana* leaves administered with 2 mL at 100 mg/kg BW and suspended in carboxymethyl cellulose and 0.5% Na solutions. Group 6 (K6) was the test group treated with 2 mL of chitosan nanoparticles of *P. americana* leaves at a dose of 100 mg/kg BW. Drug and preparations were given for 7 days after the 16% NaCl solution was given, and above-normal blood pressure was obtained. When the blood pressure decreased, a 2 mL blood sample was taken for the ACE inhibition test, nitric oxide (NO) level test, and diuretic effect test. In all rats, a 16% NaCl solution was continuously administered to maintain hypertension at the time of treatment.

### ACE inhibitor and NO serum test

Blood samples of 2 mL were taken through orbitals after a decrease in blood pressure. Then, samples were centrifuged at a speed of 10,000 × g at 4°C for 15 minutes to separate the blood from the serum. Serum ACE, nitrite, and nitrate levels were tested based on the instructions contained in each kit. ACE inhibitors and NO level tests were performed using enzyme-linked immunosorbent assay (ELISA) using the ACE ELISA kit and NO Assay kit from Thermo Fisher Scientific, Waltham, Massachusetts, United States of America.

### Diuretic effect test

The diuretic effect test uses individual metabolic cages that separated urine from rat feces to avoid interference with urine volume measurement. Urine volume was collected 24 h after measuring blood pressure, and based on *CODA*™ mouse rat tail-cuff system, blood pressure returned to normal levels.

### Data analysis

In this study, univariate and bivariate analyses were performed to obtain data on the results of the phytochemical test, SBP and DBP changes, urine volume, results of ACE inhibition test with IC _50_ parameters, and serum levels of nitric oxide. In the bivariate analysis, data processing included editing, coding, data entry, cleaning, and tabulating. Univariate analysis was carried out in each variable. In this study, values were obtained and described as mean SBP before and after treatment, mean DBP before and after treatment, rate of blood pressure reduction, urine volume after 24 h, mean percentage of ACE inhibitors (IC _50_), and mean serum level of nitric oxide. Results of the univariate analysis are presented in distribution tables, graphs, and narratives for further information. Urine volume was calculated using the diuretic activity index (DAI). Bivariate analysis was used to examine differences between groups using the SPSS 22.0 program (RRID:SCR_002865). Kruskal–Wallis test was performed for non-parametric analysis of data without normal distribution, followed by the Mann–Whitney test, while one-way analysis of variance (ANOVA) test was performed as parametric test of data with normal distribution. All groups were tested for normality with the Shapiro–Wilk test and homogeneity test to determine variance (homogeneous or heterogeneous) of data for each group.

### Study setting

This study was conducted from April to August 2019 in various laboratories, including UNDIP FSM Ecology and Biosystematics Laboratory for *P. americana* leaf determination; Texture Analysis Laboratory UNDIP Integrated Laboratory of UPT for the manufacture of extracts and activity tests for antioxidants and mineral compounds; Food Technology Laboratory of Soegijapranata Catholic University (UNIKA) for quercetin compound testing; Laboratory of Pharmacology and Toxicology Division of Pharmacology and Clinical Pharmacy, Faculty of Pharmacy, UGM, for measuring DBP, SBP, and diuretic effects; and Biochemistry Laboratory, Biotechnology Studies Center, Inter-University Center (PAU), UGM, for ELISA ACE inhibitor test and an assay of nitrite and nitrate levels.

## Results

### Determination/identification of *P. americana* leaves

Plants used were determined to be true avocados plant ( *P. americana*), with the following information:

*Kingdom: Plantae*

*Subkingdom: Tracheobionta (vascular plant)*

*Super Division: Spermatophyta (produces seeds)*

*Division: Magnoliophyta (flowering plant)*

*Class: Magnoliopsid–Dycotyledoneae (two dicots)*

*Sub Class: -*

*Order: Laurales*

*Family: Lauracea*

*Genus: Persea*

*Species: Persea americana* Mill.

### Results of *P. americana* leaf extraction

**Figure 1.**
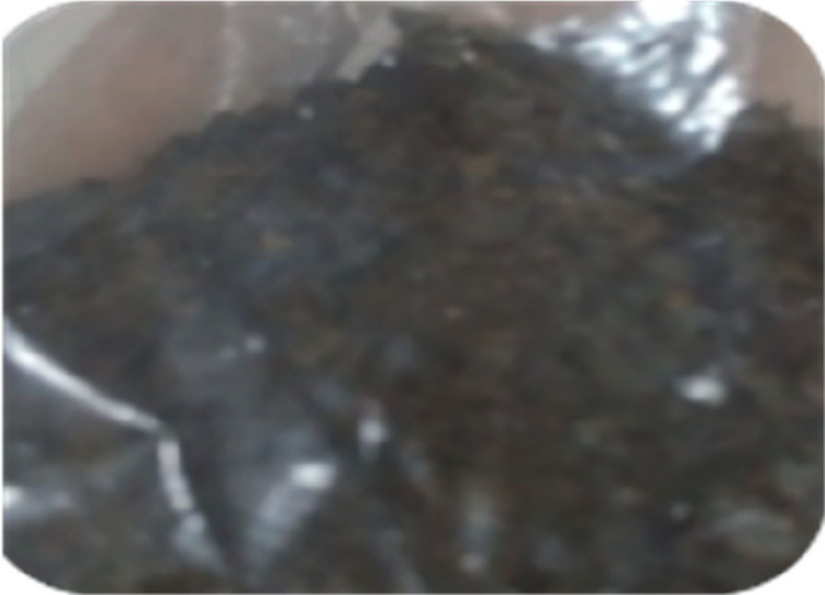
Solid preparation of *P. americana* Mill leaves extracts.

Processing of *P. americana* leaves was conducted at the texture analysis laboratory of the UNDIP Integrated Laboratory. Figure 1 shows solid preparation of *P. americana* leaf extracts. As shown in Figure 1, extracts were obtained from solid preparations of *P. americana* leaves. This preparation was also used as a sample for the phytochemical test to determine levels of quercetin, mineral compound, and antioxidant activity.

### Phytochemical test results

#### Quantification of Quercetin

High-performance liquid chromatography (HPLC) was performed for the quercetin test of samples of *P. americana* leaf extract. Based on the result, samples contained quercetin of 1129.597 ppm.

#### Antioxidant activity

The analysis of antioxidant activity using the 2,2-diphenyl-1-picrylhydrazyl method showed that the antioxidant activity at IC _50_ was 44.734 ppm.

#### Mineral compound analysis

**Figure 2.**
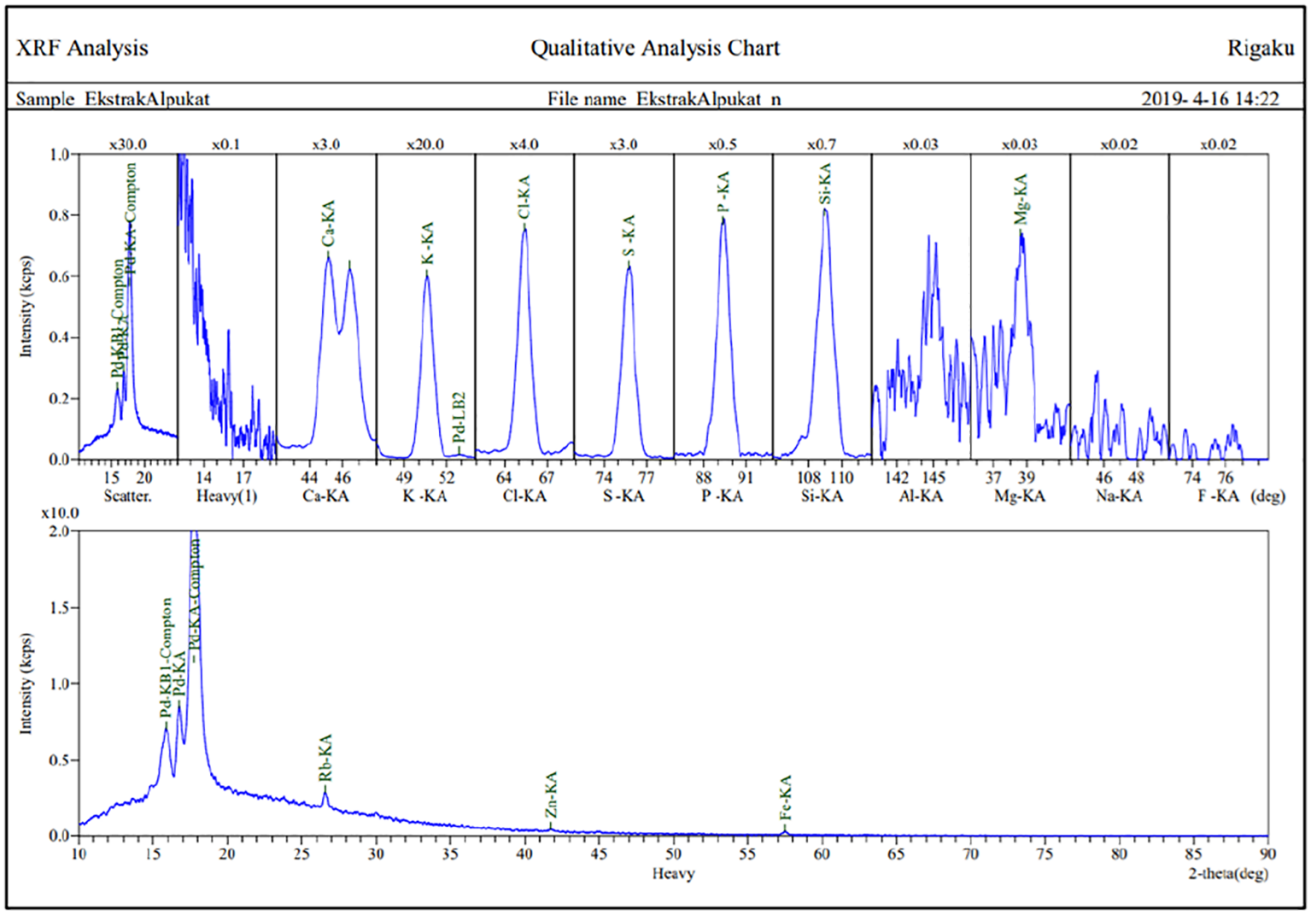
Graph of mineral compound.

Figure 2 presents detailed test results of the analysis of mineral compounds using the 2,2′-azinobis-3-ethyl benzothiazolin–sulfonic acid method. Levels of mineral compounds in leaf extracts ( *P. americana*) tested using the 2,2′-azinobis-3-ethyl benzothiazolin–sulfonic acid method are shown in Table 1. Table 1 shows that the extract contains 10 mineral compounds including potassium as the highest content, chlorine, sulfur, silicone, calcium, phosphorus, magnesium, iron, rubidium, and zinc.

**Table 1.** Mineral compounds in *P. americana* leaves extract.

No	Component	Symbol	Result (%)
**1.**	Potassium	K	1.0100
**2.**	Chlorine	Cl	0.1210
**3.**	Sulfur	S	0.1150
**4.**	Silicone	Si	0.1030
**5.**	Calcium	Ca	0.1020
**6.**	Phosphorus	P	0.0470
**7.**	Magnesium	Mg	0.0171
**8.**	Iron	Fe	0.0068
**9.**	Rubidium	Rb	0.0042
**10.**	Zinc	Zn	0.0016

#### Hypertension subjects

Four white male Wistar rats were utilised in each group, not including the six Wistar rats excluded in the analysis due to death. Rats’ blood pressure was measured before and after treatment with a 16% NaCl solution continuously administered for 14 days, followed by 7 days of receiving the preparations. Blood pressure was measured non-invasively using a *CODA*™ mouse rat tail-cuff system. To induce above-normal blood pressure, rats were administered with a 16% NaCl solution for 14 days. Furthermore, furosemide treatment (K3), *P. americana* leaf extract (K4), *P. americana* leaf extract nanoparticles (K5), and chitosan nanoparticles (K6) were administered orally in each group. In all groups except K1, the 16% NaCl solution was administered to maintain hypertensive conditions. Blood pressure was measured again after 7 days of receiving the preparations (day 22). When the rats’ blood pressure decreased, 2 mL blood samples were taken to test the activity of ACE inhibitors and measure serum levels of nitric oxide. Subsequently, rats were left to stand for 3 h after collection of blood samples and put into individual metabolic cages for 24 h to check for the diuretic effect between treatment groups.

#### Changes in SBP, DBP, urine volume, and DAI

In this study, two stages of SBP and DBP changes were observed. In the first stage, SBP and DBP were measured on day 0 (before the experiment). Then, K2–K6 rats were administered 16% NaCl solution to induce hypertension. At the second stage (day 15 to 21), K2–K6 rats still received 16% NaCl solution, while K3–K6 were given treatment according to their respective groups for 7 days. The SBP and DBP was re-measured on day 22.

**Figure 3.**
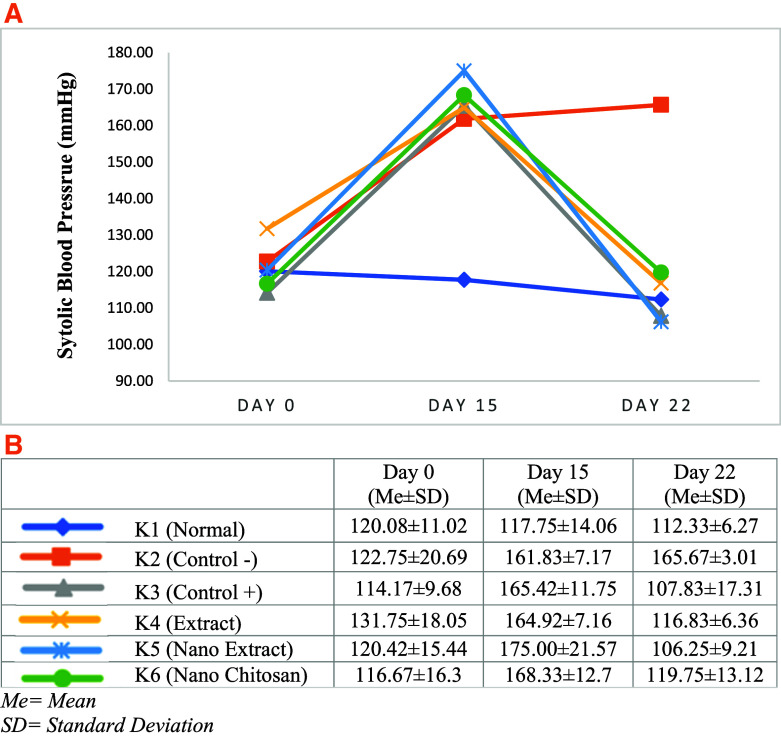
Change trend of systolic blood pressure (Mean) before the experiment and after treatment (A), Blood pressure (Standard Deviation) before the experiment and after treatment (B).

**Figure 4.**
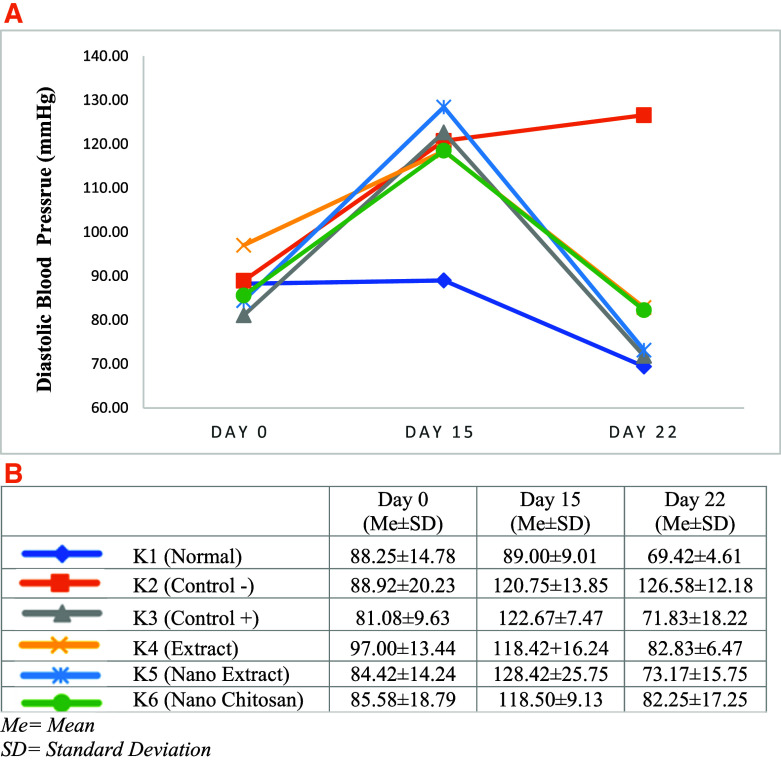
Change trends in diastolic blood pressure (Mean) before the experiment and after treatment (A), Blood pressure (Standard Deviation) before the experiment and after treatment (B).

Based on Figure 3 and Figure 4, the mean of SBP and DBP in K3–K6 groups declined after the treatment (days 22) compared with day 15. The greatest decrease of SBP and DBP was experienced by the K5 group with the *P. americana* leaf extract nanoparticles intervention by 68.75 mmHg (175.00 mmHg to 106.25 mmHg) and 55.25 mmHg (128.42 mmHg to 73.17 mmHg), respectively, followed by K3 in which the changes of SBP and DBP were 57.58 mmHg (165.42 mmHg to 107.83 mmHg), and 50.83 mmHg (122.67 mmHg to 71.83 mmHg). On the other hand, the SBP and DBP in K4 groups declined by 48.08 mmHg (164.92 mmHg to 116.83 mmHg), and 35.58 mmHg (118.42 mmHg to 82.83 mmHg), while the SBP and DBP changes in K6 group were 48.58 mmHg (168.33 mmHg to 119.75 mmHg), and 36.25 mmHg (118.50 mmHg to 82.25 mmHg), respectively ( Figure 3 and Figure 4).

#### Increased urine volume

**Figure 5.**
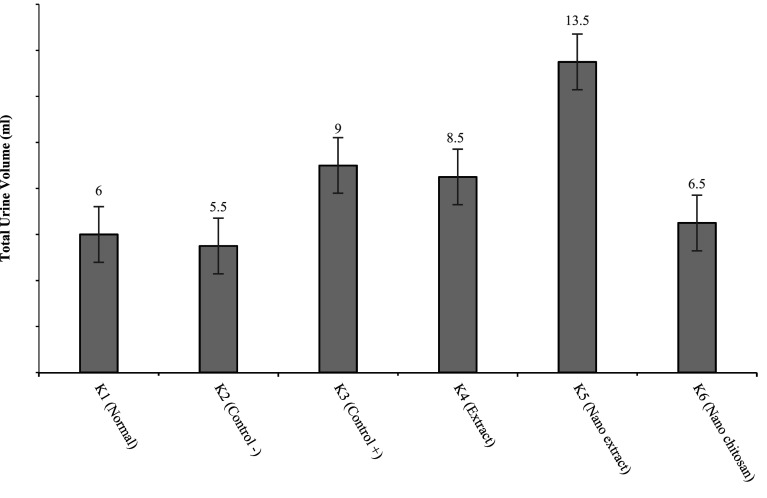
Urine volume after 24 h of treatment.

Diuretic effects can be seen through the increase in urine volume between the groups treated by measuring the urine volume after all rats were placed in individual metabolic cages for 24 h. Figure 5 shows that K2 and K6 have lower urine volumes than K3–K5. This is because K2 and K6 do not contain active substances that can increase urine volume, causing little urine excretion.

**Figure 6.**
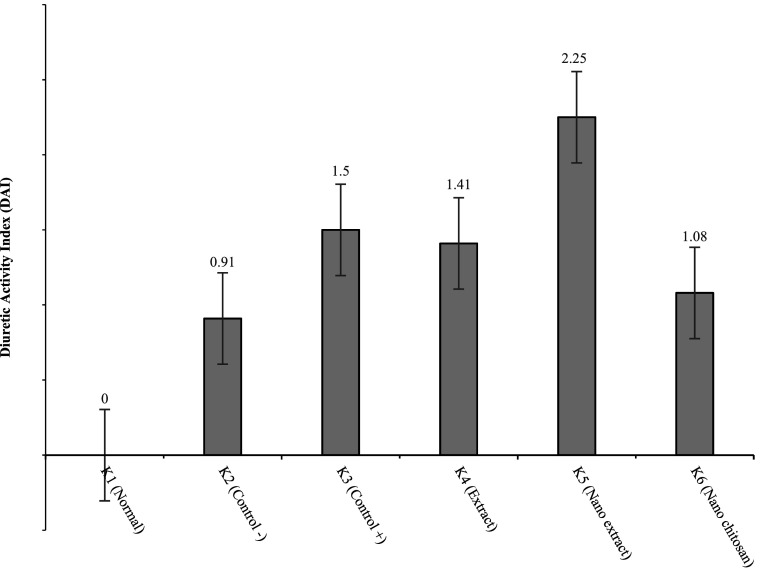
Graph of diuretic activity index.

Figure 5 reveals that the groups with the highest to lowest diuretic activities were K5, K3, K4, K6, and K2, respectively. K5 had the highest DAI value of 2.25, which indicates that K5 had high diuretic activity ( Figure 6).

#### Test results of ACE inhibitions, nitrite oxide, and nitrate oxide levels

**Figure 7.**
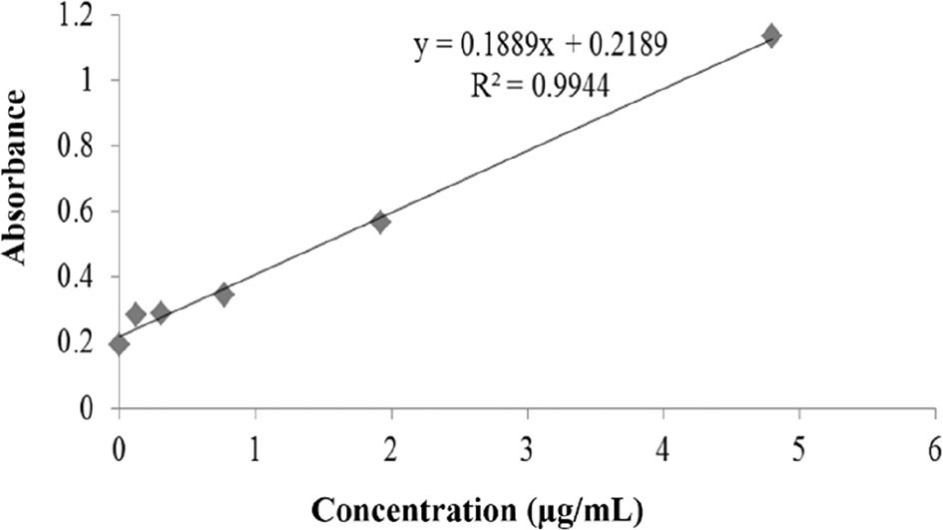
Results of the ACE inhibition test.

The results of the ACE inhibition test using the ELISA method in rat serum are shown in Figure 7. The ACE inhibition test was conducted according to the instructions on the ACE ELISA kit. Validation of the ACE inhibition test shows the performance required in the ACE ELISA kit. The calibration graph shows a linear equation (line y=0.1889x+0.2189) and linearity value (R ^2^=0.9944) ( Figure 7). The mean ACE inhibitions in the ELISA test are presented in Table 2. As depicted in Table 2, on mean, ACE was inhibited by the *P. americana* leaf extract and the nanoparticle extract were 60.0±12.1%, and 59.5±3%, respectively.

**Table 2.** Mean ACE inhibition, mean nitrate serum levels, and mean nitrite serum levels.

Sample	ACE inhibition		Serum nitrate		Serum nitrite	
	% ACE inhibition	Mean (μmol/L) ± SE	μmol/L	Mean (μmol/L) ± SE	μmol/L	Mean (μmol/L) ± SE
*P. americana* leaf extract (K4)	73.3	60.0±12.1	70.5	44.0±9.0	155.5	83.7±24.0
	66.6		39.8		62.3	
	76.4		30.9		52.8	
	24.1		34.6		64.2	
Nanoparticle extract (K5)	51.8	59.5±3.3	43.8	41.1±11.5	81.6	81.0±23.2
	56.1		72.2		146.3	
	64.7		19.6		42.5	
	65.6		28.8		53.7	
Chitosan nanoparticles (K6)	53.9	63.8±5.7	39.5	60.4±26.0	60.2	120.1±66.3
	80.4		34.0		58.0	
	59.4		29.6		43.2	
	61.6		138.4		318.8	

**Figure 8.**
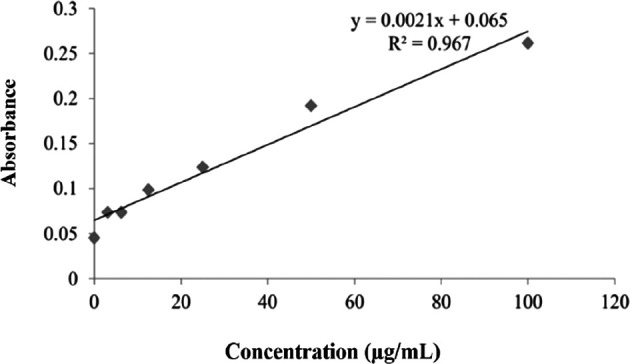
Serum level of nitrates.

The results of measuring nitric oxide serum level using the ELISA method were divided into two, namely, nitrate level test and nitrite level. Concentrations of serum nitrate are shown in Figure 8. The nitrate level test graph was conducted according to the NO Assay kit instructions. Validation of the nitrate test shows that the test followed the requirements in the NO Assay kit. The calibration graph shows a linear equation (line y=0.0021x+0.065) and a linearity value of R ^2^ of 0.967 ( Figure 8). The mean levels of nitrate oxide are shown in Table 2. As shown in Table 2, chitosan nanoparticles (K6) caused the highest mean serum nitrate with 60.4±26.0 μmol/L, while the mean serum nitrate as a result of *P. americana* leaf extract (K4), and nanoparticles of *P. americana* leaf extract (K5) were 44.0±9.0 μmol/L, and 41.1±11.5 μmol/L, respectively.

**Figure 9.**
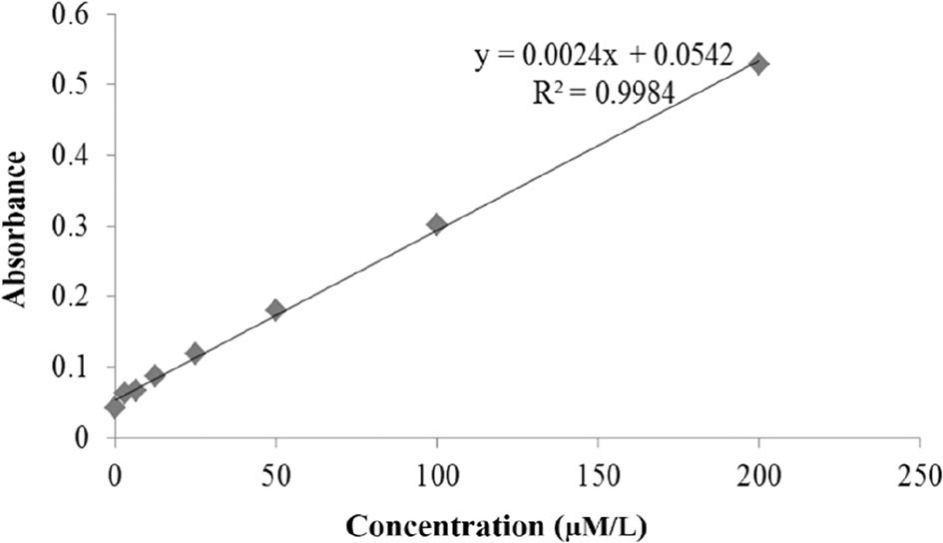
Result of the nitrite level test.

Nitrite levels using the ELISA method are shown in Figure 9. As presented in Figure 9, the graph of the nitrite level test was created correctly according to the NO Assay kit instructions. Validation of the nitrite test shows that the test followed the requirements of the NO Assay kit. The calibration graph shows a linear equation (line y=0.0024x+0.0542) and a linearity value (R ^2^=0.9984) ( Figure 9). The mean levels of nitrite oxide are shown in Table 2. Table 2 reveals that chitosan nanoparticles (K6) resulted in the highest mean of serum nitrite with 120.1±66.3 μmol/L, while the mean of serum nitrite was led by *P. americana* leaf extract (K4), and nanoparticle of *P. americana* leaf extract (K5) were 83.7±24.0 μmol/L, and 81.0±23.2 μmol/L, sequentially.

#### Bivariate analysis

Results of the data normality test showed that only the SBP group had a normal data distribution. Table 3 presents results of the data normality test of group data for SBP, DBP, and urine volume.

**Table 3.** Group data normality test for SBP, DBP, and urine volume.

Treatment group	SBP	DBP	Volume urine
**K1 (Normal)**	0.682	0.875	0.683
**K2 (Control−)**	0.230	0.596	0.001
**K3 (Control +)**	0.805	0.875	0.001
**K4 (Extract)**	0.377	0.141	0.001
**K5 (Nano extract)**	0.945	0.260	0.272
**K6 (Nano chitosan)**	0.771	0.046	0.001

Based on Table 3, the data normality test used the Shapiro–Wilk test since the number of samples was less than 50. The data distribution was considered normal if p>0.05 so the SBP group had normal data distribution. Since the data distribution was normal, the data variant test was further tested using the variant homogeneity test. Results of the variant homogeneity test in the SBP group aim to determine which variants come from the same variant and do not show significant differences from one another. Significance was set at p<0.05 for different data variants. The homogeneity test results of SBP data variance had a p-value of 0.001 (p<0.05), which indicated that the data variance was different, so a one-way ANOVA Tamhane’s test was performed.

#### SBP differences between groups

The one-way ANOVA test in the SBP group gained p-value of 0.000 (p<0.05). The test results showed significant difference in SBP between the treatment groups ( Table 4). Results of *post hoc* Tamhane analysis for comparison of SBP between groups obtained significance value of p<0.05 between K1 and K2, K2 and K4, K2 and K5, and K2 and K6, which showed significant differences in SBP between two treatment groups ( Table 5).

**Table 4.** SBP between groups tested using one–way ANOVA.

Treatment group	n	Mean±SE	Minimum	Maximum	p-value
**K1 (Normal)**	4	112.3±3.1	105.7	119.3	0.000 *
**K2 (Control −)**	4	165.7±1.5	161.3	168.0	
**K3 (Control +)**	4	116.2±12.6	87.7	144.0	
**K4 (Extract)**	4	124.8±2.0	120.3	128.7	
**K5 (Nano extract)**	4	105.8±4.3	95.7	116.3	
**K6 (Nano chitosan)**	4	112.0±5.0	109.3	133.7	

* Significant. The measurement was conducted post treatment on the Day 22.

**Table 5.** SBP comparison between groups analyzed using *post hoc* Tamhane’s.

Treatment group	Mean±SE	Minimum	Maximum	p-value
**K1 and K2**	**−**53.3±3.4	**−**73.6	**−**33.0	0.001 *
**K2 and K3**	49.5±12.7	**−**54.9	153.9	0.353
**K2 and K4**	40.9±2.5	28.5	53.2	0.000 *
**K2 and K5**	59.8±4.5	29.9	89.7	0.004 *
**K2 and K6**	43.6±5.2	7.2	80.0	0.027 *
**K3 and K4**	**−**8.5±12.7	**−**110.7	93.5	1.000
**K3 and K5**	10.3±13.3	**−**79.5	100.2	1.000
**K3 and K6**	**−**5.8±13.5	**−**92.0	80.3	1.000
**K4 and K5**	18.9±4.7	**−**8.6	46.4	0.189
**K4 and K6**	2.7±5.3	**−**30.9	36.4	1.000
**K5 and K6**	**−**16.1±6.5	**−**47.1	14.8	0.533

* Significant. The measurement was conducted post treatment on the Day 22.

#### Differences in DBP between groups

Owing to the non-normal data distribution, the Kruskal–Wallis test was used to determine differences in DBP between groups. The analysis gained value of p=0.03, which means there was a significant difference in DBP between the treatment groups ( Table 6). Thereafter, a *post hoc* Mann–Whitney test was performed to determine differences between treatment groups. In the *post hoc* Mann–Whitney test comparing DBP between groups, the p-value was <0.05, which indicated differences in DBP between K1 and K2, K2 and K3, K2 and K4, K2 and K5, and K2 and K6 ( Table 7).

**Table 6.** DBP differences between groups.

Treatment group	Mean±SE	Median	Minimum	Maximum	p-value
**K1 (Normal)**	69.4±2.3	70.0	63.3	74.3	0.03 *
**K2 (Control −)**	126.5±6.0	129.3	110.0	137.6	
**K3 (Control +)**	77.6±11.7	78.3	50.3	103.6	
**K4 (Extract)**	82.5±3.1	84.8	73.3	87.3	
**K5 (Nano extract)**	73.5±8.0	78.6	50.3	86.3	
**K6 (Nano chitosan)**	83.3±9.0	90.5	56.3	96.0	

* Significant. The measurement was conducted post treatment on the Day 22.

**Table 7.** DBP comparison analyzed using *post hoc* Mann–Whitney.

Treatment group	p-value
**K1 and K2**	0.02 *
**K2 and K3**	0.02 *
**K2 and K4**	0.02 *
**K2 and K5**	0.02 *
**K2 and K6**	0.02 *
**K3 and K4**	1.00
**K3 and K5**	0.66
**K3 and K6**	0.56
**K4 and K5**	0.38
**K4 and K6**	0.24
**K5 and K6**	0.14

* Significant. The measurement was conducted post treatment on the Day 22.

#### Urine volume test

From the Kruskal–Wallis test results on urine volume, we obtained a p-value of 0.002 (p<0.05). The value indicated a significant difference in the increase in urine volume between the treatment groups ( Table 8). Furthermore, the *post hoc* Mann–Whitney test was utilized to examine differences between the treatment groups. Results of the *post hoc* Mann–Whitney test (p<0.05) showed a significant difference in the increase in urine volume between K2 and K3, K2 and K4, K2 and K5, K3 and K5, K3 and K6, K4 and K5, K4 and K6, and K5 and K6 ( Table 9).

**Table 8.** Kruskal–Wallis test on urine volume.

Treatment group	n	Mean±SE	Median	Minimum	Maximum	p-value
**K1 (Normal)**	4	1.5±0.20	1.50	1.00	2.00	0.002 *
**K2 (Control −)**	4	1.3±0.12	1.50	1.00	1.50	
**K3 (Control +)**	4	2.2±0.25	2.00	2.00	3.00	
**K4 (Extract)**	4	2.1±0.12	2.00	2.00	2.50	
**K5 (Nano extract)**	4	3.3±0.23	3.25	3.00	4.00	
**K6 (Nano chitosan)**	4	1.6±0.12	1.50	1.50	2.00	

* Significant. The urine volume increase measurements were performed 24 hours after the decrease in blood pressure on Day 22.

**Table 9.** *Post hoc* Mann Whitney on urine volume increase.

Treatment group	p-value
**K2 and K3**	0.01 *
**K2 and K4**	0.01 *
**K2 and K5**	0.01 *
**K2 and K6**	0.18
**K3 and K4**	0.85
**K3 and K5**	0.03 *
**K3 and K6**	0.04 *
**K4 and K5**	0.01 *
**K4 and K6**	0.04 *
**K5 and K6**	0.01 *

* Significant. The urine volume increase measurements were performed 24 hours after the decrease in blood pressure on Day 22.

## Discussion

One of the factors that cause primary hypertension is excessive salt intake and increased circulation of natriuretic hormone, which inhibits intracellular sodium transport and results in an increase in extracellular fluid volume due to salt accumulation in the body. ^27^ ^,^ ^28^ The decrease in SBP and DBP can be influenced by the contents of *P. americana* leaf extract, namely, flavonoids and quercetin compound. ^7^ ^,^ ^14^ Flavonoids and quercetin can reduce SBP and DBP because these compounds can inhibit ACE, which converts angiotensin I to angiotensin II causing vasoconstrictions and thus increasing blood pressure. Quercetin compound can inhibit ACE activity by 60.0%, increasing endothelial relaxation and widening blood vessels, so blood is smoothly supplied to the heart. Inhibition of ACE activity by *P. americana* leaf extract proves that the bioactivity of quercetin compound is functionally excellent for antihypertensives. ^7^ ^,^ ^11^ ^,^ ^29^

This study showed that the extract inhibited ACE by >50%. ACE needs to be inhibited because it converts angiotensin I to angiotensin II as a vasoconstrictor of precapillary arterioles and postcapillary venules. Inhibited production of angiotensin II can lead to lowering the blood pressure. ^7^ ^,^ ^10^ ^,^ ^24^ ^,^ ^30^ In addition, the study reveals that the extract contains potassium, and magnesium. Potassium afffect the blood pressure lowering through improvement of endothelial function, NO release, and vasodilatation. ^31^ Magnesium plays role to blood pressure regulation partly via its sympatholytic property and vasodilator action. Beside, the antihypertensive effect is related to calcium channel blockage, increases in NO, and better endothelial function. Increasing NO level in serum lead to relaxing vascular smooth muscle cells and reducing blood pressure. ^31^ ^,^ ^32^

The current study reveals that the extract resulted in serum nitrite and nitrite means of >40 μmol/L and >80 μmol/L. Regarding the antihypertensive mechanism of action, an increase in nitrate and nitrite levels is important because they are related to blood pressure; a lower blood pressure indicates higher NO levels in the blood or vice versa. ^17^ ^,^ ^33^ Nitrates and nitrites increase level lead to the capacity dilating of the arteries. ^34^ The antihypertensive effects are related to reduced NADPH oxidase-derived oxidative stress. NADPH oxidase has an important role in the nitroso-redox imbalance. ^35^ Nitrite also works as a precursor for the systemic generation of vasodilatory nitric oxide, and exogenous nitrate administration leads to lowering blood pressure. ^36^

Moreover, SBP and DBP decreased because *P. americana* leaf extract has high antioxidant activity. ^23^ Antioxidant activity is related to Nitric Oxide Synthase (NOS) levels where quercetin can increase NOS activity in endothelial cells acting on arteries by stimulating or activating endothelium-derived relaxing factor, causing vasodilation of endothelial cells. ^4^ ^,^ ^17^ The results of this study showed that *P. americana* leaf extract was effective in reducing SBP and DBP until normal blood pressure is attained. Compared with furosemide, *P. americana* leaf extract showed lower SBP and DBP-reducing effect, but it was only slightly different. The nanoparticle method significantly reduces the frequency of doses related to pharmacodynamics but optimizes the efficacy on target organs related to pharmacokinetics. ^37^ ^–^ ^41^

The results of this study also show that the use of *P. americana* leaf extract decreased SBP and DBP owing to the diuretic activity of quercetin compound, as shown in the increased urine volume measured after treatment. Diuretics are compounds or drugs that can increase urine volume. ^42^ ^,^ ^43^ Flavonoid compounds and quercetin increase urine volume by inhibiting sodium reabsorption, triggering its discharge. As a result, the kidneys quickly remove waste products from the body. ^4^ ^,^ ^44^

There were some limitations to this study. Firstly, the potential zeta value in the *P. americana* Mill leaf extract nanoparticles is not yet stable, and there was no measurement of the potential zeta value for nano chitosan. Secondly, rats weighing less than 200 grams are difficult to adjust to the holder and rubber on *CODA*™ mouse rat tail-cuff system, causing blood pressure measurements to be more difficult than rats weighing over 200 grams. Thirdly, measuring the blood pressure of rats on day 0 before treatment was difficult because the rats had not adapted to the *CODA*™ mouse rat tail-cuff system causing the measurement time to be longer. Besides, the 16% NaCl induction group was placed in one cage where rats under stress conditions due to hypertension should be separated into individual cages to avoid death in rats.

This research was only limited to the potential of extracts and nanoparticles of *P. americana* Mill leaf chitosan extract. Further research is needed to test the toxicity of the kidneys and liver of test animals and on LD50 to determine the optimum dose of *P. americana* Mill leaf extract nanoparticles as a curative antihypertensive. Furthermore, additional research is needed to manufacture more modern nanoparticles such as capsules or tablets to produce products that can be applied to the public.

## Conclusion

The use of *P. americana* leaf extract is effective in reducing SBP and DBP and thus helps achieve normal blood pressure. Though its blood pressure-reducing effect is lower than that of modern medicine (furosemide), the nanoparticles extract had the most significant result in lowering SBP and DBP. Administration of *P. americana* leaf extract significantly increased urine volume in tested rats.

## Author contributions

**Sutiningsih D**: Conceptualization, Investigation, Resources, Project Administration, Visualization, Writing – Original Draft Preparation, Writing – Review & Editing; **Sari DP**: Investigation, Data Curation, Formal Analysis; **Adi MS**: Methodology, Supervision, Formal Analysis; **Hadi M**: Methodology, Formal Analysis, Validation; **Azzahra NA**: Writing – Original Draft Preparation.

## Data availability

### Underlying data

Figshare: Effect of Avocado Leaves on Systolic, Diastolic Blood Pressure, Urine Volume, and Diuretic Activity Index on Wistar Rats. https://doi.org/10.6084/m9.figshare.20390463.v3. ^45^

This project contains the following underlying data:

• SBP and DBP.xlsx (Mean diastolic blood pressure (DBP) before and after administration of 16% NaCl; and mean diastolic blood pressure (DBP) after administration of extract, extract nanoparticles, and chitosan nanoparticles).
• Urine volume and DAI.xlsx (Table. Total urine volume and diuretic activity index (DAI) after 24 hours).
• Figure of preparations.docx (Figure. Preparation of chitosan nanoparticles ethanol extract of *P. Americana* Mill leaves; and Figure. Preparation of chitosan nanoparticles).
• *P. americana* Mill leaf extract nanoparticles.pdf (Figure. Distribution graph of extract nanoparticle size)
• Chitosan nanoparticles.pdf (Figure. Distribution graph of chitosan nanoparticle size).
• Weight of Wistar rats.xlsx (Table. Mean weight of Wistar Rats Day 0 and Day 15).
• EOS.pdf (Graph of EOS Plot and Mobility Distribution of Zeta Potential)
• PVT.pdf (Peak Value Table of extract nanoparticle)

### Reporting guidelines

Figshare: ARRIVE checklist for [Effectiveness of avocado leaf extract ( *Persea americana* Mill.) as antihypertensive]. https://doi.org/10.6084/m9.figshare.20764855.v2. ^46^

Data are available under the terms of the Creative Commons Attribution 4.0 International license (CC-BY 4.0).
