# Supplementary material for: Effectiveness of avocado leaf extract ( Persea americana Mill.) as antihypertensive
Source: F1000Res. 2023 Nov 13;11:1100. Originally published 2022 Sep 27. [Version 2] doi: 10.12688/f1000research.124643.2 (PMC12409261; doi:10.12688/f1000research.124643.2)
Supplement: Peer review history

# Reviewer report: Reviewer response for version 2

**DOI:** 10.5256/f1000research.156550.r225713

**Published:** 2025-09-03

**Authors:** Dian Kresnadipayana

**Affiliations:** 1 Setia Budi University Central Java, Surakarta, Indonesia

This manuscript presents a well-designed and well-executed experimental study on the antihypertensive effects of avocado leaf extract and its nanoparticle formulation in salt-induced hypertensive Wistar rats. The topic is highly relevant and timely, considering the growing global interest in natural and plant-based therapies as alternatives or complements to conventional treatments. The study is methodologically sound, the data are clearly presented, and the conclusions are well supported by the results.

 The writing is clear and coherent, making the manuscript easy to follow. The introduction provides a solid rationale for the study, the methods are described with sufficient detail to ensure reproducibility, and the results are presented logically and with appropriate interpretation. Importantly, the discussion places the findings within the broader scientific context while maintaining focus on the unique contribution of this work.

 Overall, the manuscript makes a valuable and meaningful contribution to the field. The scientific quality, clarity, and relevance are of a standard suitable for indexing as it stands. I recommend acceptance of this manuscript without the need for further revision.

Is the work clearly and accurately presented and does it cite the current literature?

Yes

If applicable, is the statistical analysis and its interpretation appropriate?

Yes

Are all the source data underlying the results available to ensure full reproducibility?

Yes

Is the study design appropriate and is the work technically sound?

Yes

Are the conclusions drawn adequately supported by the results?

Yes

Are sufficient details of methods and analysis provided to allow replication by others?

Partly

Reviewer Expertise:

Biochemistry, Toxicology, Organic Chemistry.

I confirm that I have read this submission and believe that I have an appropriate level of expertise to confirm that it is of an acceptable scientific standard.

# Reviewer report: Reviewer response for version 2

**DOI:** 10.5256/f1000research.156550.r222816

**Published:** 2023-12-14

**Authors:** Azlini Ismail

**Affiliations:** 1 Department of Fundamental Dental and Medical Sciences, International Islamic University Malaysia, Kuantan, Pahang, Malaysia

All comments have been addressed by the authors.

Is the work clearly and accurately presented and does it cite the current literature?

Partly

If applicable, is the statistical analysis and its interpretation appropriate?

Partly

Are all the source data underlying the results available to ensure full reproducibility?

Yes

Is the study design appropriate and is the work technically sound?

Partly

Are the conclusions drawn adequately supported by the results?

Partly

Are sufficient details of methods and analysis provided to allow replication by others?

Yes

Reviewer Expertise:

Cardiovascular research, pharmacognosy, pharmacology, antihypertensive study

I confirm that I have read this submission and believe that I have an appropriate level of expertise to confirm that it is of an acceptable scientific standard.

# Reviewer report: Reviewer response for version 1

**DOI:** 10.5256/f1000research.136859.r180334

**Published:** 2023-07-12

**Authors:** Azlini Ismail

**Affiliations:** 1 Department of Fundamental Dental and Medical Sciences, International Islamic University Malaysia, Kuantan, Pahang, Malaysia

This is a research article on the effect of avocado leaf extract as antihypertensive. The article has potential to be indexed with following modifications.

**Abstract:**

1. The Introduction part of the abstract needs to rephrased in order to highlight that novelty of this study on the effect of avocado left extract on salt-induced hypertensive Wistar rats.
2. Kindly omit the objective to see the differences in effectiveness of modern drugs and the plant extract, and replace it with " *to study the effectiveness of avocado leaf extract on salt-induced hypertensive Wistar rats.*"
3. The results section need to be re-written to accurately reflect the actual findings. The SBP and DBP values do not have the SD or SE. The statistical significance for each test should be omitted, and to be replaced with the important findings from each part of the study.
4. The presence of quercetion in the extract does not guarantee that the decrement of blood pressure was due to the quercetin. Thus, the following statement needs to re-worded.
  " *The decrease in blood pressure occurred because the extract contained a quercetin compound discovered by the high-performance liquid chromatography (HPLC) method of 1129.597 ppm.*"

**Introduction:**

1. Please elaborate further on this statement; " *Hypertension is an increase in blood pressure **measured twice in resting conditions**,....* "
2. Please rephrase the following statement which is a bit confusing, " *Management of hypertension is essential because high blood pressure is a cardiovascular risk factor and **a primary clinical sign of hypertension control.*** "
3. Please rephrase the following statement which is a bit confusing, " *The body may not be able to tolerate the appropriate drug levels, **therefore cannot necessarily completely cure the disease, as the safety level of hypertension drugs must be maintained chronically.** *"
4. Quercetin is a single compound, thus it is suggested to remove the word "compounds" in the text.
5. Please consider the following suggestion: " *The Wistar rat, which has been bred at the Wistar Institute since 1906, is one of the most widely used **animal model** in biomedical research. ^11^ *"
6. Need to use the citation as per usual scientific writing. Kindly modify the following:
  Auwal's research (2017)....E.g., A previous study by Auwal et al. (2017) demonstrated that...
  Please modify accordingly for the following:
  Mariangela's (2011) research.....
  Yuan's (2012) study......
  Chong's (2014) study......
7. " *This study is relevant because results of various studies have confirmed the effectiveness of P. americana leaves in treating hypertension. ^3^^,^^7^^,^^8^^,^^21^^,^* ^*22"* ^
  With regard to the above statement, the sentence has to be modified as it indicates that the previous studies already have confirmed the effectiveness of avocado in treating hypertension. This would question the necessity of conducting this research. It is suggested to modify this sentence and highlight the gap of study that this study has fulfilled.

**Methods**

1. Kindly remove the research question and hypothesis as these are not usually mentioned in journal article.
2. Is it using the *CODA*™ mouse rat tail-cuff system? Please write in full. 
3. Please spell in full, the word 'CMC'.
4. Kindly change the heading " ***Quercetin levels" into "Quantification of quercetin"***
5. Kindly change the heading " ***Mineral compound sample" into "Mineral compound analysis"***
6. Please include standard deviation (DS) or standard error (SE) for the mean (average) readings in the text under the sections, ***Changes in SBP, DBP, urine volume, and DAI' *** and ***'Increased urine volume section. *** Please also include SD or SE in the Figure 3 and 4. This is necessary as the results should be an average reading of the four replicates.
7. Kindly replace 'mice' with 'rats' under the ***Increased urine volume section.***
8. Please adjust the position of values on Figure 5 and 6 so that they will not overlap with the error bars.
9. Table 2 has to be modified such that only one average (mean) value with the SD or SE for % ACE inhibition, serum level of nitrates and serum level of nitrites were listed for each group. 
10. Need to clearly indicate the SBP, DBP, urine volume and urine volume increase in Table 4,5,6, 7, 8, and 9. These readings were taken post-treatment or during treatment? If post-treatment, on what day (i.e. D22 etc.)?
11. The findings for serum nitrate, nitrite and ACE inhibition are not impactful as there were no values obtained for normal, non-treated Wistar rats, or the untreated hypertensive rats for comparison.

**Discussion**

1. Kindly rephrase... " *ACE needs to be inhibited because it acts as a vasoconstrictor of blood vessels, causing hypertension*" as angiotensin II is the vasoconstrictor, produced by the conversion from Angiotensin I by ACE, not by itself is a vasoconstrictor.
2. It is not that accurate to mention that potassium and magnesium act as antihypertensive agents, however it is suggested to mention the important role that they played in managing high blood pressure. 
3. Need to provide further explaination on the relation between nitrate and nitrite levels with the level of blood pressure. 
4. Please spell NOS in full for the first-time appearance in text.
5. It is better to avoid using the statement of " *Herbal medicines have safer side effects than modern drugs.*" as it is not always true in all conditions.
6. It is suggested to remove some words to avoid confusion. The new suggested statement is: " *Flavonoid compounds and quercetin increase urine volume by inhibiting sodium reabsorption, triggering its discharge.*" 

**Conclusion**

1. The conclusion should be directly related to the findings from this study. Therefore, I suggest to omit the following statement: " *As regards the mechanism of antihypertensive action, the difference is unclear; thus, it is more effective to use extracts of P. americana leaves, which contain natural compounds.*"

Is the work clearly and accurately presented and does it cite the current literature?

Partly

If applicable, is the statistical analysis and its interpretation appropriate?

Partly

Are all the source data underlying the results available to ensure full reproducibility?

Yes

Is the study design appropriate and is the work technically sound?

Partly

Are the conclusions drawn adequately supported by the results?

Partly

Are sufficient details of methods and analysis provided to allow replication by others?

Yes

Reviewer Expertise:

Cardiovascular research, pharmacognosy, pharmacology, antihypertensive study

I confirm that I have read this submission and believe that I have an appropriate level of expertise to confirm that it is of an acceptable scientific standard, however I have significant reservations, as outlined above.

# Response

**Published:** 2023-09-24

**Authors:** Dwi Sutiningsih

**Affiliations:** Diponegoro University, Indonesia

Thank you very much for the suggestion to improve our articles. We have addressed the issues based on the suggestions.

**Abstract**

 1. Have done in the revised manuscript (the latest version)

 2. Have done in the revised manuscript (the latest version)

 3. Have done in the revised manuscript (the latest version)

 4. Have done in the revised manuscript (the latest version)

**Introduction**

 1. Have done in the revised manuscript (the latest version)

 2. Have done in the revised manuscript (the latest version)

 3. Have done in the revised manuscript (the latest version)

 4. Have done in the revised manuscript (the latest version)

 5. Have done in the revised manuscript (the latest version)

 6. Have done in the revised manuscript (the latest version)

 7. Have done in the revised manuscript (the latest version)

**Method**

 1. Have done in the revised manuscript (the latest version)

 2. Have done in the revised manuscript (the latest version)

 3. Have done in the revised manuscript (the latest version)

 4. Have done in the revised manuscript (the latest version)

 5. Have done in the revised manuscript (the latest version)

 6. Have done in the revised manuscript (the latest version)

 7. Have done in the revised manuscript (the latest version)

 8. Have done in the revised manuscript (the latest version)

 9. The table has been included SD or SE in the manuscript before the revision and after the revision.

 10. The SBP and DBP Measurements in Tables 4, 5, 6, and 7 were conducted post-treatment on Day 22. The urine volume increase measurements in Tables 8 and 9 were performed 24 hours after the decrease in blood pressure on Day 22. The revision is displayed below each table.

 11. Yes, the findings for serum nitrate, nitrite and ACE inhibition were only to *P. americana* leaf extract (K4), Nanoparticle extract (K5), and Chitosan nanoparticles (K6), and were not to normal, non-treated Wistar rats, or the untreated hypertensive rats for comparison (K1).

**Discussion**

 1. Have done in the revised manuscript (the latest version)

 2. Have done in the revised manuscript (the latest version)

 3. Have done in the revised manuscript (the latest version)

 4. Have done in the revised manuscript (the latest version)

 5. Have done in the revised manuscript (the latest version)

 6. Have done in the revised manuscript (the latest version)

**Conclusion**

 Thank you very much for the suggestion. We have omitted the related statement.
